# An Automated Method for Identifying Voids and Severe Loosening in GPR Images

**DOI:** 10.3390/jimaging11080255

**Published:** 2025-07-30

**Authors:** Ze Chai, Zicheng Wang, Zeshan Xu, Ziyu Feng, Yafeng Zhao

**Affiliations:** 1 School of Computer and Control Engineering, Northeast Forestry University, Harbin 150040, China; chaichai123@nefu.edu.cn (Z.C.); 2024122604@nefu.edu.cn (Z.F.); 2 Sichuan Bright Engineering Quality Inspection Co., Ltd., Chengdu 610015, China; 17883693536@163.com

**Keywords:** ground-penetrating radar, void, severe soil loosening, FKS-GPR Dataset, deep learning

## Abstract

This paper proposes a novel automatic recognition method for distinguishing voids and severe loosening in road structures based on features of ground-penetrating radar (GPR) B-scan images. By analyzing differences in image texture, the intensity and clarity of top reflection interfaces, and the regularity of internal waveforms, a set of discriminative features is constructed. Based on these features, we develop the FKS-GPR dataset, a high-quality, manually annotated GPR dataset collected from real road environments, covering diverse and complex background conditions. Compared to datasets based on simulations, FKS-GPR offers higher practical relevance. An improved ACF-YOLO network is then designed for automatic detection, and the experimental results show that the proposed method achieves superior accuracy and robustness, validating its effectiveness and engineering applicability.

## 1. Introduction

The safety and durability of road structures are fundamental to ensuring the stable operation of transportation systems. Among the factors threatening these structures, subsurface structural defects—particularly voids and severe soil loosening—are two typical concealed anomalies that pose significant risks to road service life and driving safety [1]. Voids refer to underground cavities within localized structural regions that can drastically weaken the load-bearing capacity of the road. Severe loosening, on the other hand, describes a substantial reduction in soil or pavement compaction, typically caused by weakened cohesion or abnormally increased porosity, leading to functional degradation and reduced structural stability. Although these two types of defects differ markedly in physical origin and engineering impact, they often exhibit highly similar signal patterns in ground-penetrating radar (GPR) B-scan images. This similarity presents a major challenge for their automatic identification and differentiation [2].

Currently, most research in subsurface defect detection focuses on identifying a single type of defect or performing coarse assessments of defect presence. Fine-grained classification between easily confused anomalies, such as voids and severe loosening, remains underexplored. Existing methods largely rely on expert interpretation or traditional signal processing techniques, which offer limited accuracy, adaptability, and automation, falling short of the demands for intelligent detection in complex road environments [3,4].

In recent years, breakthroughs in deep learning have driven its application in the automatic detection of underground defects. For example, Kang et al. proposed a convolutional neural network system based on 3D GPR data, combined with a beam-tracking background suppression algorithm, to automatically detect voids beneath urban roads. This method demonstrated promising visibility and detection performance in experiments, indicating potential for engineering applications. Nevertheless, the application of deep learning to GPR image interpretation remains hindered by a critical bottleneck: the lack of high-quality, field-acquired B-scan datasets that reflect realistic road conditions. This limitation greatly restricts model generalizability and robustness under complex backgrounds [5,6,7].

Most current studies rely on simulated or synthetic images for training and validation. While these methods may perform well under ideal conditions, their adaptability in real-world environments is limited. For instance, Zhou et al. [8] proposed a method that uses wavelet decomposition to fuse measured and synthetic images for dataset construction, improving anomaly detection performance. However, the dataset was primarily simulation-based and lacked extensive real-world validation. Similarly, the DMRF-UNet proposed by Dai et al. [9], which incorporates residual structures and transfer learning, showed improved performance on synthetic and some measured images. Yet it only enabled coarse “defect/non-defect” classification, without exploring the structural differences between voids and loosening, thereby limiting its engineering applicability and precision scalability.

In summary, current research on subsurface defect detection faces several key limitations. First, the classification granularity remains insufficient—most existing studies concentrate on detecting a single type of defect, with limited efforts dedicated to analyzing and distinguishing between visually similar types such as voids and severe soil loosening. Second, many deep learning approaches rely heavily on simulated or synthetic data for training, which often fails to capture the complexity, variability, and noise present in real-world road environments. Third, there is a notable lack of high-quality, field-acquired B-scan datasets that are both representative and diverse, severely restricting the generalization ability and robustness of trained models when deployed in practical engineering scenarios. Lastly, the recognition scope of most current methods is limited to binary classifications (defect vs. non-defect), with little emphasis on fine-grained identification of defect severity or specific defect types, which is essential for targeted maintenance and structural assessment.

Therefore, in response to the challenges posed by complex real-world road environments, there is an urgent need to conduct fine-grained recognition research targeting voids and severe soil loosening. This includes building high-quality GPR image datasets grounded in real engineering scenarios and developing deep learning detection frameworks with strong discriminative capability and robustness. These efforts are essential to advance underground defect detection from coarse identification to precise recognition. The main contributions of this paper are as follows:

1. First systematic analysis of deep image-level differences between voids and loosened areas: To address the challenge of highly similar signal patterns between voids and severe loosening in GPR B-scan images, this study conducts a systematic analysis from three key dimensions: image texture structure, the intensity and clarity of the top reflection interface, and the regularity of internal reflection waveforms. This provides a theoretical foundation for accurate defect classification.
2. Development of a high-quality GPR dataset reflecting real-world scenarios: We construct the FKS-GPR dataset, which includes a wide range of void and severe loosening samples collected from actual road environments. This fills a critical gap in available measured datasets with complex background features, enhancing the generalizability of models in engineering applications.
3. Proposal of an improved ACF-YOLO (Attention–Convolution Fusion YOLO) framework for underground defect detection: This paper introduces an enhanced ACF-YOLO architecture that integrates attention mechanisms and refined feature fusion strategies to achieve high-precision automatic detection of voids and severe loosening, significantly improving detection performance in complex scenarios.

## 2. Related Work

### 2.1. Principle of Ground-Penetrating Radar (GPR) Detection

Ground-penetrating radar (GPR) emits high-frequency electromagnetic waves in the form of broadband pulses through an antenna. When these electromagnetic waves encounter a target object, reflection or transmission occurs, and the reflected signals are received by the receiving antenna. As the waves propagate through different media, their propagation paths, electromagnetic field intensity, and waveforms vary due to changes in the dielectric properties and structural characteristics of the media. By collecting, processing, and analyzing these time domain waveforms, the spatial location and structural condition of underground interfaces or target objects can be determined. In road inspection, particular attention is given to the difference in dielectric constants between the media on either side of the reflecting interface, as this directly affects the phase and amplitude of the reflected waves [10]. The reflection coefficient *R* is expressed by the following formula:

(1) $$R=\frac{\sqrt{\varepsilon_{r1}}-\sqrt{\varepsilon_{r2}}}{\sqrt{\varepsilon_{r1}}+\sqrt{\varepsilon_{r2}}}$$

In the above formula, $\varepsilon_{r1}$ denotes the relative dielectric constant of the upper medium, and $\varepsilon_{r2}$ denotes that of the lower medium. When the dielectric constant of the subsurface medium is greater than that of the pavement structure, electromagnetic waves traveling from the pavement into the subsurface medium result in a negative reflection coefficient *R*, causing the reflected wave to have a phase opposite to that of the incident wave. Conversely, when the dielectric constant of the subsurface medium is less than that of the pavement structure, the reflection coefficient *R* is positive, and the reflected wave’s phase aligns with the incident wave. The greater the difference between the dielectric constants of the subsurface material and the pavement structure, the larger the absolute value of the reflection coefficient *R* is expressed by Equation (1), as shown above, leading to a stronger reflected wave amplitude at the interface. Conversely, if the dielectric constants are similar, the absolute value of *R* is small, resulting in a weaker reflected wave amplitude. This difference in dielectric constants directly influences the reflection characteristics of the GPR signal, thereby affecting the detection and imaging quality of the target objects.

### 2.2. Principle of Voids and Severe Soil Loosening Identification Based on B-Scan Image Features

**Figure 1:**
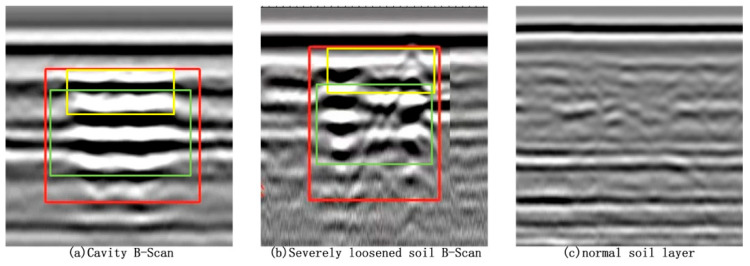
Comparison of B-scan images of voids, severe soil loosening, and normal soil layers.

Although both voids and severe soil loosening manifest as low-dielectric constant regions within subsurface structures and can easily be confused on ground-penetrating radar (GPR) B-scan images, significant differences exist between them in terms of electromagnetic wave propagation paths, reflection characteristics, and image manifestations. To address this, the present study proposes a recognition approach based on detailed features extracted from B-scan images to effectively discriminate between void and severe loosening areas. Specifically, the analysis is conducted along three dimensions: image texture structure, top reflection interface features, and internal reflection waveform regularity, as illustrated in Figure 1. The red boxes highlight regions corresponding to void samples in (a) and severe loosening samples in (b).

At the image texture structure level, as shown in Figure 1, voids generally contain air or extremely dry materials with homogeneous structures and simple electromagnetic wave reflection paths, resulting in concentrated echoes. This appears in the image as clear boundaries, regular textures, and distinct reflection contours. In contrast, severe loosening regions exhibit heterogeneous internal media with disordered pore distributions, causing significant electromagnetic wave multipath scattering. Consequently, the images show chaotic echoes resembling “cloudy” or “honeycomb” patterns, with markedly poorer texture continuity and regularity. Therefore, void images typically demonstrate higher order and regularity, whereas severe loosening images exhibit disordered and chaotic structural characteristics.

Secondly, from the perspective of the top reflection interface (indicated by the yellow rectangle in Figure 1), voids exhibit a strong reflection due to the significant dielectric contrast between air and foundation materials. This appears as a continuous, high-brightness, and sharply contoured horizontal reflection band in the image. In contrast, although severe loosening areas also involve dielectric differences, their top reflection interfaces are relatively blurred because the structure is not completely fractured. This manifests as unclear boundaries, smooth grayscale transitions, and weaker reflection brightness. This distinction provides a valuable reference for practical discrimination.

Regarding the regularity of internal reflection waveforms (shown in the green rectangle in Figure 1), void interiors are largely free of impurities, allowing radar waves to reflect repeatedly between upper and lower interfaces, producing regular multiple echo stripes. These appear as vertically symmetrical and periodically distinct band structures in the image. Conversely, severe loosening areas have fragmented internal structures and weakened cohesion, leading to complex reflection paths and significant waveform distortion. The images show inconsistent waveform phases, increased interference, and disordered stripes. Thus, voids exhibit clear, regular waveforms, while loosening areas display chaotic and blurred structural features.

To further improve discrimination reliability, this study includes B-scan samples of normal soil layers for comparison (Figure 1c). Normal soil is uniform and compact internally, with stable radar wave propagation paths. Its images reveal uniform, continuous reflections with smooth grayscale gradients and natural boundary transitions. Compared to voids, normal soil lacks strong top reflection bands and regular multiple echoes; compared to severe loosening, it shows clearer texture, more uniform reflection distribution, and absence of obvious scattering noise. This makes normal soil images relatively neutral and stable, serving as an important baseline for distinguishing voids and loosening.

In conclusion, although voids and severe loosening share some physical similarities, analyzing their multidimensional features—texture structure, top interface characteristics, and waveform regularity—in GPR B-scan images, combined with comparison to normal soil, enables clear and reliable identification and differentiation. Based on this theoretical analysis, this study further collected and constructed the accurately labeled, structurally diverse FKS-GPR dataset, covering voids, severe loosening, and normal soil samples. In subsequent sections, an improved ACF-YOLO deep recognition network is designed and implemented for automated identification experiments, thoroughly validating the effectiveness and practicality of the proposed discrimination approach.

### 2.3. YOLOv8 Object Detection Network

Since Redmon and Farhadi [11] introduced YOLOv1, object detection algorithms have attracted widespread attention. YOLOv7 achieved excellent performance on standard datasets, but its detection speed and memory usage still struggle to meet the strict real-time requirements, especially in engineering scenarios demanding high efficiency, such as rail defect detection. To further improve model performance, YOLOv8 incorporates multiple structural optimizations based on YOLOv7, including replacing the original C3 modules with more expressive C2f structures, adjusting channel numbers across different model scales, introducing a decoupled detection head, and adopting an anchor-free design philosophy, thus achieving significant advancements in accuracy and flexibility.

Nonetheless, YOLOv8 still faces certain limitations in detecting targets of varying scales in complex backgrounds, being susceptible to noise-induced missed detections, leaving room for overall accuracy improvement. To address these issues, this paper proposes a lightweight object detection network named ACF-YOLO, with detailed improvements and model design presented in Section 4: Methods.

## 3. Collection and Construction of the FKS-GPR Dataset

To evaluate the adaptability and recognition performance of the proposed object detection model in complex real-world road environments, this study constructs the FKS-GPR dataset based on field-acquired radar images. The data collection was jointly conducted by Northeast Forestry University and the ground-penetrating radar (GPR) testing team led by expert Zeshan Xu. Field experiments were carried out across all road sections within Dingbian County, Yulin City, Shaanxi Province. The following sections provide a detailed overview of the data acquisition environment, equipment configuration, and data processing workflow.

### 3.1. Data Acquisition Environment and Soil Characteristics

**Figure 2:**
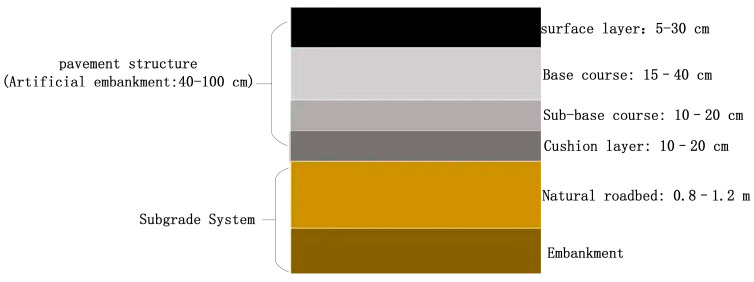
Main layers of the road structure.

The data acquisition for this study was conducted in Dingbian County, Yulin City, Shaanxi Province, with the primary engineering objective of detecting typical structural subsurface defects—namely, underground voids and severe soil loosening—within county-level roadways. The road structure in this region can be broadly divided into two main layers: the upper pavement structure and the lower subgrade system. The pavement structure consists of several layers arranged from top to bottom: the surface layer (5–30 cm thick), the base layer (15–40 cm thick), the sub-base layer (10–20 cm thick), and the cushion layer (10–20 cm thick). These layers are bonded together using cementitious materials or asphalt concrete to collectively bear traffic loads and provide a stable driving surface. The subgrade system comprises a natural or backfilled roadbed (typically 0.8–1.2 m thick) and an artificial embankment (40–100 cm thick). On either side, drainage layers or slope protection structures are typically installed to accommodate terrain variations and maintain structural stability. The detailed configuration is illustrated in Figure 2.

The subsurface media in this region are highly complex and mainly include typical road structure materials such as sand, gravel, asphalt concrete, and cement concrete. Significant differences exist in physical parameters such as compaction degree, moisture content, and particle gradation among different structural layers, resulting in spatial heterogeneity and dynamic variation in their relative permittivity. These factors directly affect the propagation speed, reflection intensity, and energy attenuation characteristics of electromagnetic waves within the medium, which in turn determine the identifiability and stability of signals in ground-penetrating radar (GPR) images. In particular, within the subgrade region, the complex composition of fill materials, uneven compaction, and frequent fluctuations in moisture content often induce strong multipath reflections and scattering interference, leading to highly unstable and nonlinear radar echo signals. This significantly increases the difficulty of interpreting B-scan images and places higher demands on the robustness of subsequent defect recognition algorithms [12,13,14].

To enhance the environmental diversity and spatial coverage of the dataset, this study conducted multiple rounds of radar data acquisition under different time periods (day and night) and various climatic conditions (high temperature, low temperature, dry, and humid). Especially under alternating dry–wet road conditions and heavy traffic flow, external environmental factors such as vehicle vibration and electromagnetic interference caused significant variation in background noise characteristics across different times and regions. In the radar signal spectrum, the low-frequency range (100–500 MHz) is primarily influenced by large-scale structural disturbances (e.g., uneven compaction, cracks, and voids), manifesting as widespread strong background reflections. In contrast, the high-frequency range (500 MHz–2 GHz) is more susceptible to scattering interference from fine-grained material distribution, rebar mesh structures, and underground pipelines, leading to enhanced local signal oscillations and blurred textures in images, further increasing sample variability and recognition difficulty.

These complex and variable geological conditions and acquisition environments not only enrich the diversity and realism of the FKS-GPR dataset but also provide a more rigorous and engineering-relevant experimental foundation for subsequent evaluations of model generalization and robustness. Compared with traditional studies that rely on simulated data, the dataset and acquisition strategy developed in this study offer greater practical value for real-world engineering applications, effectively supporting the stable detection and accurate identification of voids and severe loosening defects under diverse scenarios and interference conditions.

### 3.2. Equipment Configuration and Acquisition Parameters

This study conducted large-scale ground-penetrating radar (GPR) data acquisition across various types of road environments, utilizing radar systems with advanced features such as high-frequency operation, dual polarization, and multi-channel capabilities, to meet the requirements for high-precision target identification in complex road structures.

**Figure 3:**
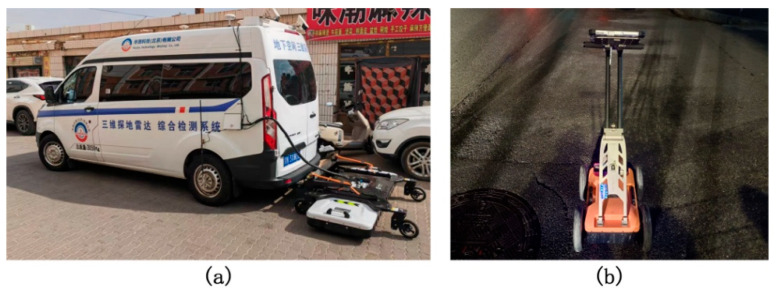
(**a**) Vehicle-mounted ground-penetrating radar systems operating on roads. (**b**) The operation of hand-propelled ground-penetrating radar instruments on roads.

For wide roads and highways, large-area coverage data acquisition was primarily carried out using the vehicle-mounted GPR system SIR-4000, manufactured by Geophysical Survey Systems, Inc. (GSSI), Nashua, NH, USA, as shown in Figure 3a. This system supports a wide frequency range from 50 MHz to 2.6 GHz. In this study, 900 MHz and 1.2 GHz antennas were selected to target medium-shallow and medium-deep subsurface structures, respectively, achieving a practical balance between penetration depth and image resolution. The system features a dual-polarization design, equipped with horizontal polarization (H-Pol) and vertical polarization (V-Pol) channels, enabling simultaneous transmission and reception of radar signals. This enhances sensitivity and identification capability for targets in different orientations.

During field acquisition, the vehicle-mounted system moved steadily along the road surface at approximately 30 km/h. The sampling interval was set to 20 cm, with a time window of 25 ns, effectively controlling spatial resolution while ensuring sufficient detection depth. The system was equipped with dual triggering mechanisms via GPS and an onboard odometer to ensure accurate alignment between radar signals and spatial position. At each sampling point, 3000 time samples were recorded, and B-scan image data were simultaneously acquired in both HH and VV polarization modes. The front and rear antenna channels corresponded to HH and VV polarizations, effectively functioning as two independent radar systems working in tandem. Although cross-polarization (HV/VH) data could not be acquired simultaneously, the available dual-polarization information already provided high-quality input for subsequent texture analysis, multi-channel data fusion, and target classification [15].

To further enhance data quality, the antennas were rigidly mounted beneath the vehicle using a dedicated bracket and shielded with microwave-absorbing materials to suppress electromagnetic interference from the vehicle and the surrounding environment, thereby significantly improving the signal-to-noise ratio. For narrow road sections or areas inaccessible to large vehicles, supplementary data collection was conducted using a handheld ZRY-30 model 400 MHz GPR device, as shown in Figure 3b. This portable device allowed operators to obtain close-range, high-density data in complex terrain, making it well-suited for detailed detection of edge areas and fine structural features. The handheld radar was operated by professionally trained personnel, moving at a steady, low speed along predetermined paths with uniform spacing to ensure complete coverage and consistency within the target areas.

To enhance the usability of the data and ensure stability in subsequent image analysis, all raw radar data underwent standardized preprocessing after acquisition. This included DC drift correction, ground coupling wave suppression, gain balancing, ringing effect elimination, band-pass filtering, and background signal removal. These procedures effectively suppressed artifacts caused by system responses and environmental background noise, significantly improving the signal quality and structural clarity of the resulting B-scan images. As a result, a reliable data foundation was established for the subsequent identification of voids and severe looseness based on image features.

### 3.3. FKS-GPR Dataset

To promote the practical application of ground-penetrating radar (GPR) in automated road defect detection, this study conducted large-scale in situ radar data acquisition and target annotation based on the previously described environments and equipment configurations. A high-quality, engineering-representative, and complex-scene-oriented object detection dataset was constructed, named the FKS-GPR dataset. This dataset focuses on two of the most common and easily confused subsurface hazards in road structures—underground voids and severe soil looseness. It also incorporates complex background interference, various subsurface material types, and dual-polarization radar imaging, providing strong data diversity and broad applicability for supporting model training and generalization performance evaluation.

During the dataset construction process, a large number of 2D B-scan images and partial 3D radar imaging data collected by both vehicle-mounted and handheld radar systems were used. A team of experts in subsurface structure detection, led by Professor Xu Zeshan, was invited to perform manual interpretation and detailed annotation. The expert group accurately marked the locations and boundaries of underground voids and severe looseness by comprehensively analyzing key indicators such as texture features in the images, the intensity of the top reflection band, and the regularity of internal echo waveforms, while leveraging 3D imaging results to enhance spatial consistency and coherence in target identification.

**Figure 4:**
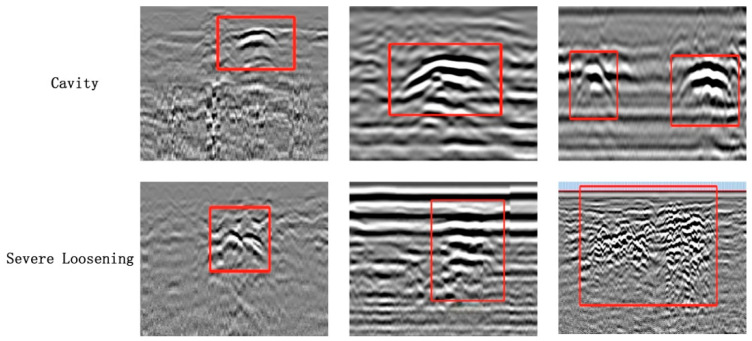
Typical samples from the FKS-GPR dataset.

As a result, 107 voids and 129 severely loosened regions were identified and annotated within the real radar data collected across the main roads of Dingbian County, forming a total of 202 high-quality B-scan sample images containing 236 valid targets. Several representative examples are shown in Figure 4. Given the relatively limited volume of raw data, the expert team additionally contributed early-stage measured images from the Shenyang region. After integration, an enhanced version of the B-scan dataset was formed, consisting of 350 images with 426 annotated targets in total. All images were standardized to a resolution of 640 × 256 pixels and accompanied by normalized target bounding boxes and category labels to meet the input requirements of mainstream object detection models.

**Figure 5:**
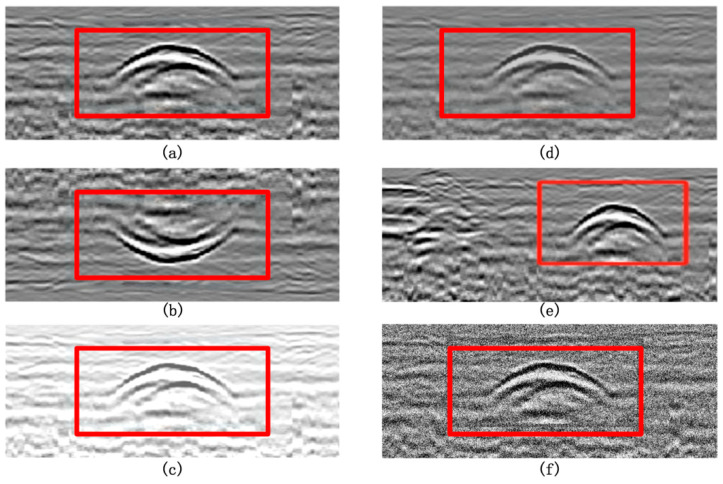
Examples of original and augmented images: (**a**) original sample; (**b**) vertical flipping; (**c**) +30% brightness perturbation; (**d**) contrast adjustment; (**e**) B-scan cropping; and (**f**) Gaussian noise addition.

To enhance the model’s robustness to varying scenarios and interference conditions, this study incorporated multiple image-level data augmentation strategies on the constructed dataset, including image rotation, flipping, brightness perturbation, local contrast adjustment, image cropping, and synthetic noise addition. While preserving the structural features of the targets, these methods effectively increased the dataset’s diversity, representativeness, and generalization capability, ultimately expanding the dataset to 2800 image samples. The composition ratio and class statistics are detailed in Table 1. The expanded FKS-GPR dataset was split into training and validation sets at an 8:2 ratio, ensuring sample independence during model evaluation. As shown in Figure 5a–f, comparisons are presented between several original samples and the corresponding augmented samples generated using different data augmentation techniques: (a) original sample; (b) vertical flipping; (c) +30% brightness perturbation; (d) contrast adjustment; (e) B-scan cropping; and (f) Gaussian noise addition. It can be observed that these augmentation methods, while preserving the fundamental structural characteristics of the targets, significantly enhance the diversity and complexity of the samples. Moreover, they effectively simulate various environmental interferences and imaging condition variations that may occur in real-world detection scenarios, thereby improving the model’s generalization ability and robustness under complex conditions.

**Figure 6:**
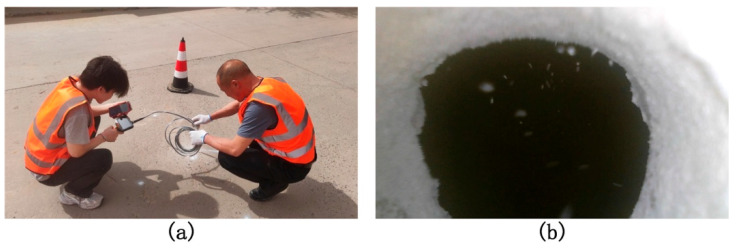
(**a**) On-site work photo; (**b**) endoscopic image of cavity.

It is noteworthy that, to verify the accuracy of expert interpretations and the reliability of data annotations, field drilling and manual inspections were conducted at selected typical sample locations within the validation set. These on-site investigations provided direct validation of the subsurface structural conditions, as illustrated in Figure 6a, which shows photographs of the field verification work, and Figure 6b, which presents the cavity image captured after on-site drilling. The comparison results reveal a high degree of consistency between the radar-detected cavities and severely loosened areas and the actual observations obtained from drilling. This not only provides robust engineering-level validation for the image interpretation methodology but also fundamentally enhances the authenticity and credibility of the FKS-GPR dataset for practical engineering applications.

The construction of this dataset also overcomes the limitations of traditional studies that overly rely on simulated data or samples collected under ideal experimental conditions. By integrating radar data acquisition with complex geological backgrounds and actual road structural characteristics, it effectively restores the radar imaging features and interference patterns encountered in real-world application scenarios. The FKS-GPR dataset demonstrates significant advantages in sample quality, scene complexity, and labeling accuracy, providing a solid and reliable data foundation for subsequent research on deep learning-based target detection methods in ground-penetrating radar images. It also provides important support for robustness evaluation and practical engineering validation of related models.

## 4. Methods

Hollows and severe soil loosening areas in ground-penetrating radar (GPR) images typically exhibit distinctive features such as blurred upper boundaries, uneven internal energy distribution, and subtle local texture disturbances. Unlike rigid targets in natural images with clear edges and shapes, these underground structural hazards often manifest as a superposition of multiple signal patterns in radar images, combining characteristics of reflected energy distribution, structural boundary variations, and texture detail differences. They possess complex imaging attributes, including non-rigid boundaries, weak target features, and strong background interference. Therefore, achieving high-precision localization of target regions under complex background interference, as well as fine-grained modeling and differentiation of their internal structural details, becomes a critical technical challenge in this study.

To address these challenges, this paper proposes an improved lightweight object detection network called ACF-YOLO, based on YOLOv8, specifically designed for the automatic identification of hollows and severe loosening in GPR images. During the network architecture design and module construction, targeted structural optimizations and feature enhancements were made, focusing on three core dimensions involved in distinguishing hollows and looseness—texture structure, reflection boundaries, and waveform regularity—effectively improving the model’s recognition accuracy and robustness in complex underground structural scenarios.

### 4.1. ACF-YOLO Module

**Figure 7:**
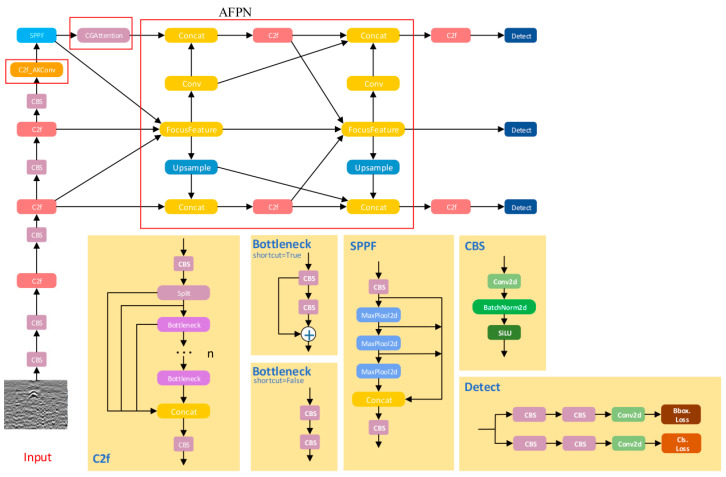
Network structure of the ACF-YOLO module.

The overall network architecture of ACF-YOLO is shown in Figure 7. Based on the basic framework of YOLOv8, the network still consists of three main modules: Backbone (feature extraction), Neck (feature fusion), and Head (detection head). To target the recognition challenges in ground-penetrating radar (GPR) B-scan images—such as interference from background noise, blurred top interfaces, and weak internal texture disturbances of hollow and severe loosening targets—targeted structural optimizations and functional enhancements were implemented.

During the multi-scale feature fusion stage, ACF-YOLO introduces an Attention-guided Feature Pyramid Network (AFPN), which applies saliency weighting mechanisms to feature maps of different scales. This effectively highlights the response features of potential target regions under strong noise backgrounds or complex geological layers, achieving clearer target perception and localization.

In the Backbone part, the network embeds a dynamic convolution module that employs adaptive deformable convolution kernels to flexibly adjust the receptive field’s shape and direction. This enables accurate extraction of critical boundary features even when facing weak reflection signals at the hollow tops or irregular edge structures, thereby enhancing modeling capability for non-rigid boundaries.

Furthermore, after the SPPF (Spatial Pyramid Pooling—Fast) module at the end of the Neck, a Channel-Guided Attention mechanism (CGAttention) is integrated. By jointly modeling the echo energy distribution and texture spectral features in radar images, this mechanism significantly improves the network’s perception of internal structural variations and local texture anomalies within severe loosening areas.

These three improved modules work synergistically within the overall network architecture, enabling ACF-YOLO to significantly enhance detection accuracy and robustness for hollow and loosening hazards in GPR images while maintaining the excellent inference efficiency of YOLOv8. This provides reliable support for intelligent recognition in complex underground structural scenarios.

### 4.2. Focused Pyramid Module

To address the common challenges in ground-penetrating radar (GPR) images—such as strong background interference, large variations in target scale, and complex, variable target shapes—this study designs an adaptive multi-scale feature focusing pyramid network (Attention-guided Feature Pyramid Network, AFPN) based on the PKIModule described in Reference [16], integrating it into the multi-scale feature fusion stage of ACF-YOLO. Compared to the traditional FPN structure, AFPN introduces an attention gating mechanism in the feature fusion path, which adaptively highlights salient features of underground hazards like cavities and severe soil loosening according to the response intensity of feature maps at different scales, while suppressing interference responses from non-target regions. Considering the significant morphological differences and random spatial distribution of these two types of targets across scales, AFPN effectively enhances the network’s overall perception capability for target regions, demonstrating stronger robustness and stability, especially under heavy noise backgrounds.

**Figure 8:**
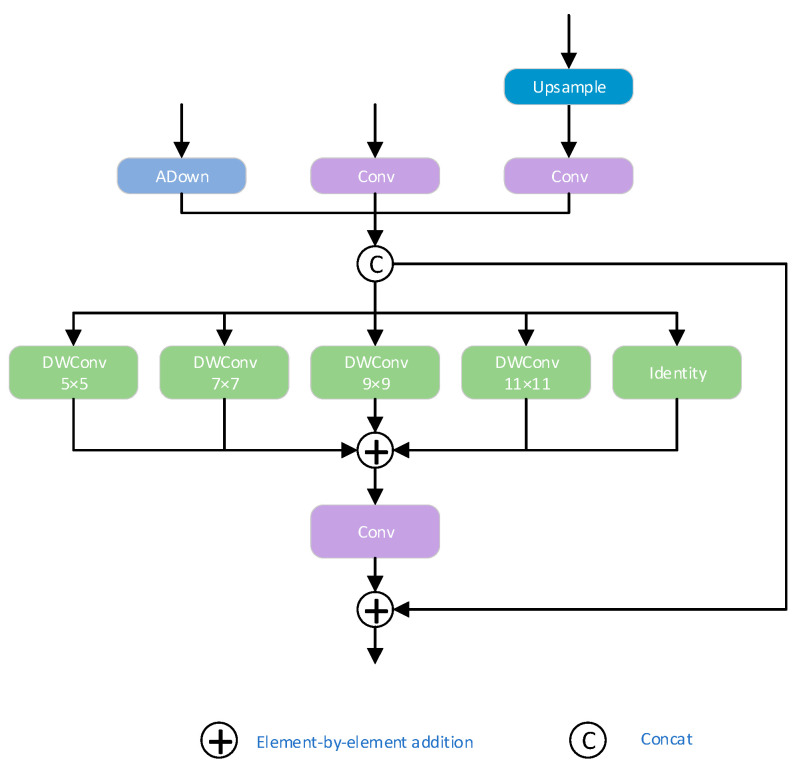
Network structure of the Focused Pyramid module.

By strengthening semantic focus at the whole-image level, AFPN significantly reduces missed detections and false positives caused by complex textured backgrounds, and provides more precise candidate regions to the subsequent detection head that relies on local fine-grained features, thereby improving overall recognition accuracy. Furthermore, the Focus Feature submodule integrated within AFPN employs an Inception-style multi-branch parallel architecture, containing three scale branches that, respectively, receive feature map inputs at different resolutions. Each branch efficiently extracts multi-scale local information via depthwise separable convolutions, fully capturing spatial scale variations in underground structures, enriching spatial information representation, and enhancing the ability to characterize fine-grained target features. Its specific structure is shown in Figure 8.

### 4.3. AKConv Module

In ground-penetrating radar (GPR) images, voids and severe soil loosening areas typically exhibit distinct physical response differences at the top reflection interface. Voids, being air-filled, have a dielectric constant significantly different from the surrounding foundation material, causing the radar waves to form strong, high-intensity reflections with clear boundaries at this interface. In contrast, severe loosening areas, whose structures are not completely broken, have relatively smaller dielectric constant differences and usually appear as weak echo bands with blurred boundaries and gradual grayscale transitions. These distinguishing features form the core basis for differentiating these two types of underground hazards.

However, in the original YOLOv8 network, the standard convolution operation used in the Backbone employs fixed kernel shapes and receptive field sizes, limiting its ability to adaptively model the top structures of different target types. Specifically, for the strong boundary structures of voids, conventional convolution can still extract relatively clear high-gradient features; but when dealing with the blurred, irregular weak echo structures of loosening areas, the rigidity of the convolution kernel and the limited local receptive field cause the feature responses to be weak, thereby increasing the risk of missed detections and false positives.

To enhance the network’s modeling capability for top reflection interfaces, this paper introduces an Adaptive Kernel Convolution module (AKConv) [17] into the key downsampling stages of the YOLOv8 Backbone. Its structure is shown in Figure 7. This module dynamically adjusts the shape, size, and receptive field of the convolution kernel based on the local structural information of the input feature map, enabling adaptive modeling of complex boundary structures. The improved network can focus more on the strong boundary reflection features when identifying voids, and expands the receptive field to better respond to weak signals when identifying severe loosening areas, significantly enhancing the model’s sensitivity and modeling effect on different top structural features.

**Figure 9:**
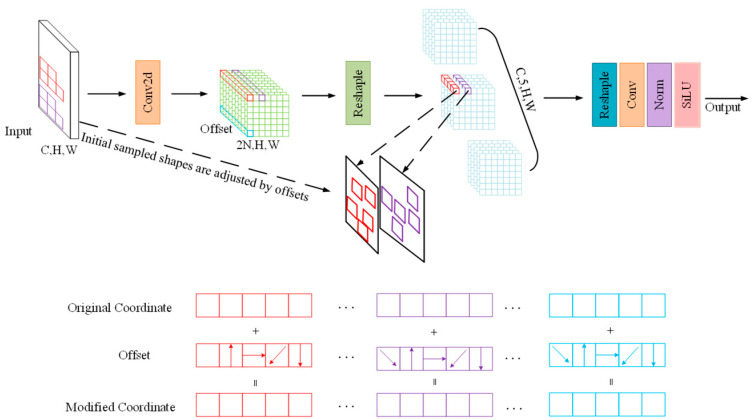
Network structure of the AKConv module.

The structure of the AKConv module is shown in Figure 9, with its core lying in the adaptive irregular sampling mechanism. Traditional convolution operations use fixed regular sampling grids; for example, the sampling grid *R* for a 3 × 3 convolution is as follows:

(2) $$R=\left\{\left(-1,-1\right)\right.,\left(-1,0\right),\ldots ,\left(0,1\right),\left.\left(1,1\right)\right\}$$

However, this regular sampling method is difficult to adapt to target regions in GPR images with significant structural differences. To address this, AKConv designs a convolution kernel coordinate generation algorithm that supports arbitrary kernel sizes. The algorithm first generates a conventional regular sampling grid, then perturbs the coordinates of the remaining sampling points to convert it into an irregular grid. These are finally concatenated to form the complete convolution sampling coordinate set, all unified with the top-left corner (0,0) as the reference origin to accommodate convolution kernels of different sizes and shapes. After defining the initial coordinates $P_{n}$ for the irregular convolution, the convolution at position $P_{0}$ is defined as follows:

(3) $$\mathrm{Conv}\left(P_{0}\right)=\sum_{P_{n}\in R}w\times x\left(P_{0}+P_{n}\right)$$

Here, *R*, *w*, and $x\left(P_{0}+P_{n}\right)$ represent the generated sampling grid, convolution parameters, and the pixel values at corresponding positions, respectively. However, convolution operations on irregular sampling grids face challenges in matching. To address this, AKConv introduces offsets to dynamically adjust the sampling shape, improving the accuracy of feature extraction. The offsets are obtained via convolution operations and have dimensions (*B*,2*N*,*H*,*W*), where *N* is the size of the convolution kernel (as shown in Figure 7, *N* = 5). These offsets are combined with the original coordinates $\left(P_{0}+P_{n}\right)$, and through interpolation and resampling, features are extracted. The features can be stacked for convolution or reshaped into a four-dimensional tensor (*C*,*N*,*H*,*W*) to extract features with specific strides and kernel sizes. During training, the AKConv module automatically learns the optimal offsets through backpropagation, enabling dynamic perception and feature enhancement of target regions with different structural characteristics. This mechanism effectively improves the network’s responsiveness to structural boundary variations while keeping the model lightweight. The experimental results demonstrate that the introduction of the AKConv module significantly enhances the model’s detection robustness and stability in complex soil backgrounds and multi-scale target scenarios. In particular, it achieves higher accuracy when identifying weak-signal loosened areas, providing a stronger foundation for subsequent feature extraction and target classification.

Although the proposed AKConv module shares conceptual similarities with existing deformable convolution techniques, such as Deformable Convolutional Networks v2 (DCNv2) [18], there are significant differences between the two in terms of design motivation, implementation, and application scenarios. DCNv2 primarily enhances spatial sampling flexibility for natural image tasks by learning continuous offsets for each sampling point, thereby improving the network’s ability to capture geometric transformations and object deformations. However, this approach has not been specifically optimized for the unique characteristics of ground-penetrating radar (GPR) images, where subsurface targets such as voids and severe soil loosening typically exhibit highly variable boundary clarity and signal intensity in reflection patterns.

It is particularly important to emphasize that the primary motivation for introducing the AKConv module lies in addressing the significant differences in reflection interface strength observed in GPR images. Specifically, void regions are characterized by strong reflections and sharp, well-defined boundaries, while severely loosened regions display weak signal reflections with blurred boundaries and gradual grayscale transitions. Therefore, AKConv dynamically adjusts sampling positions and receptive fields to maintain high sensitivity to strong boundary reflections while effectively enhancing responsiveness to weak boundary areas. In contrast, DCNv2, as a general-purpose deformable convolution method designed for natural images, does not incorporate such signal strength considerations and is more prone to missed detections or false positives when applied to GPR data with weak targets.

In comparison, the AKConv module integrates irregular sampling grid construction with offset-based dynamic deformation in a lightweight design, enabling flexible adaptation to both strong boundary reflections of voids and weak texture echoes of loosened regions. Unlike DCNv2, AKConv constrains the deformation flexibility within a reasonable range, which not only enhances feature extraction sensitivity but also ensures higher computational efficiency—an essential requirement for real-time and stable performance in practical engineering applications.

Furthermore, although DCNv2 performs well in modeling texture variations in natural images, its direct application to GPR data is suboptimal due to the weak grayscale transitions and low-contrast boundaries inherent in radar imagery. The hybrid adaptive mechanism of AKConv simultaneously strengthens the network’s perception of both high-gradient and low-gradient regions, thereby significantly improving detection accuracy and robustness in complex underground environments. The performance differences between AKConv and DCNv2 are further quantitatively analyzed in the ablation study presented in Section 5.5.

### 4.4. CG Attention Module

In ground-penetrating radar (GPR) images, cavities and severely loosened soil areas exhibit significant differences in the orderliness of internal texture structures and the regularity of reflected waveforms, which are key factors for accurate identification and differentiation of these two types of targets. Cavity regions are typically filled with air or dry media, possessing relatively uniform structures; radar wave propagation paths are stable, and reflected signals are concentrated with obvious periodicity. Thus, in the images, they manifest as orderly textures, clear boundaries, and striped multiple echoes. In contrast, severely loosened areas have disordered pore distributions and chaotic electromagnetic properties, which tend to cause multipath scattering and random interference. This results in images with chaotic, irregular textures and echo features resembling “foggy” or “honeycomb-like” diffusion, lacking clear periodic structures [19]. Although these differences in internal regularity and orderliness are crucial for distinguishing cavities from loosened areas, traditional detection networks often struggle to precisely capture and model such fine-grained distinctions, mainly due to two issues: (1) feature channels are weighted equally without accounting for the differing importance of texture and waveform regularity across channels; (2) there is a lack of spatial response mechanisms for texture perturbations and periodic structures, resulting in insufficient recognition of abnormal textures in loosened regions, which negatively affects detection accuracy.

To address these problems, this study introduces a Channel-Guided Attention mechanism (CGAttention) after the SPPF module in the YOLOv8 network to jointly model texture structural features and internal reflected waveform regularity [20]. This module combines the advantages of channel attention and spatial attention: channel attention dynamically adjusts the weights of feature channels to emphasize discriminative channel information closely related to “texture orderliness” and “waveform periodicity”; simultaneously, spatial attention enhances the network’s perception and modeling ability of local abnormal texture regions in the image. This improves recognition of the disordered textures and irregular reflections characteristic of loosened areas. During training, CGAttention adaptively strengthens feature responses for regions with strong regularity and orderly textures (such as cavities) while enhancing identification of areas with disordered textures and chaotic echoes (such as loosened soil), resulting in differentiated feature enhancement strategies. The experimental results demonstrate that integrating CGAttention enables the network to reliably detect cavities and loosened areas under complex backgrounds and high noise conditions, effectively reducing missed and false detections, and significantly improving overall detection accuracy and robustness. This provides key support for achieving highly reliable automatic identification of underground defects.

**Figure 10:**
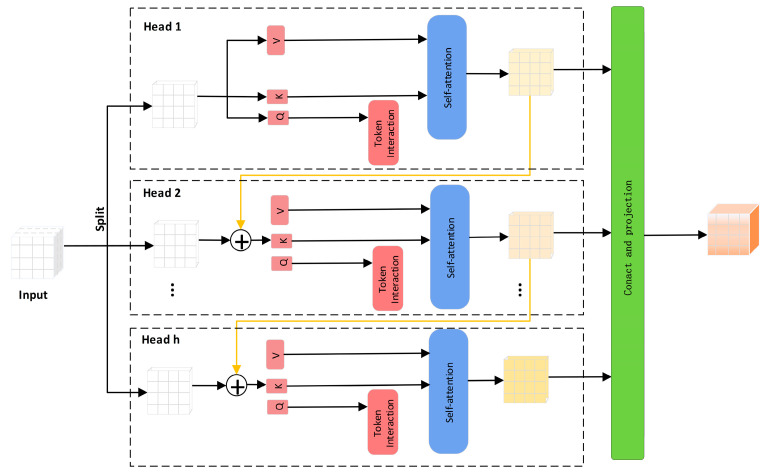
Network structure of the CGAttention module.

To further explain the role of token interaction and self-attention within the CGAttention module (as illustrated in Figure 10), the mechanism allows each token—representing a specific spatial position in the feature map—to compute self-attention scores relative to all other tokens. This design enables the model to dynamically capture both local and global dependencies between texture patterns. Specifically, the token interaction mechanism allows informative features, such as the periodic textures of cavity regions, to influence distant spatial areas of the feature map, thereby enhancing global contextual modeling. Meanwhile, the self-attention mechanism empowers the network to selectively focus on meaningful texture cues and effectively suppress irrelevant noise by dynamically adjusting feature importance across spatial locations. Through this dual mechanism, the model achieves superior capacity in distinguishing the structured, repetitive features of cavities from the irregular, chaotic reflections of loosened areas, ultimately leading to more accurate and robust detection performance.

Therefore, the CGAttention module not only provides a mechanism specifically tailored for modeling texture and energy features but also achieves substantial performance improvements over traditional architectures. It serves as an indispensable key component for distinguishing the internal characteristics of cavities and severe looseness. Its network structure is shown in Figure 10. Specifically, the CGAttention mechanism can be formulated as Equation (4):

(4) $${\tilde{X}}_{ij}=\mathrm{Attn}(X_{ij}W_{ij}^{Q},\;X_{ij}W_{ij}^{K},\;X_{ij}W_{ij}^{V})$$

(5) $${\tilde{X}}_{i+1}=\;\mathrm{Concat}[{\tilde{X}}_{ij}{]}_{j=1:h}W_{i}^{P}$$

Here, the *j*-th head computes self-attention on $X_{ij}$, which is the *j*-th split of the input features, $X_{ij}\;$= [$X_{i1}$, $X_{i2}$, …, $X_{ih}$] with 1 ≤ *j* ≤ *h*, where h is the total number of heads. The matrices, $W_{ij}^{Q}$, $W_{ij}^{K}$, and $W_{ij}^{V}$ are the projection layers that map the input features into different subspaces, and $W_{i}^{P}$ is the linear layer that projects the concatenated output features back to the input’s original dimension. The output of each head is then added sequentially to subsequent heads to progressively refine the feature representation (see Equation (6)):

(6) $$X_{ij}^{\prime }\;=\;X_{ij}\;+\;{\tilde{X}}_{i(j-1)},\;\;\;1<j<h$$

Among them, $X_{ij}^{\prime }$ is the sum of the *j*-th input split $X_{ij}$ and the output ${\tilde{X}}_{i(j-1)}$ from the (*j* − 1)-th head, as computed by Equation (5). During the self-attention computation, it replaces $X_{ij}$ as the new input feature for the *j*-th head. In addition, another token is passed through an interaction layer after the *Q* projection, enabling the self-attention mechanism to jointly capture both local and global relationships, thereby further enhancing the feature representation.

### 4.5. Visual Interpretability Analysis

**Figure 11:**
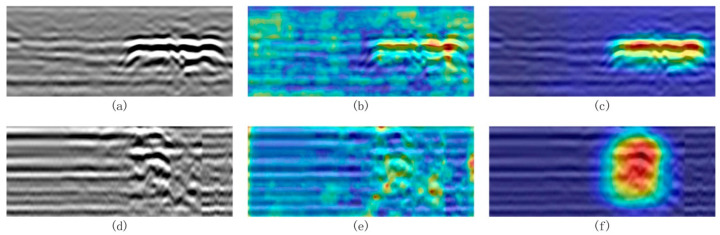
Grad-CAM visualizations of detection results for cavities and loosened soil. (**a**) Original B-scan image with cavity target. (**b**) Grad-CAM visualization of baseline YOLOv8 for cavity detection. (**c**) Grad-CAM visualization of proposed ACF-YOLO for cavity detection. (**d**) Original B-scan image with loosened soil target. (**e**) Grad-CAM visualization of baseline YOLOv8 for loosened soil detection. (**f**) Grad-CAM visualization of proposed ACF-YOLO for loosened soil detection.

To further elucidate the interpretability of the proposed model and substantiate the effectiveness of its attention mechanisms, Grad-CAM visualization was conducted on both cavity and severely loosened soil detection tasks. As illustrated in Figure 11a–f, the baseline YOLOv8 model—lacking dedicated attention modules—exhibits diffuse and occasionally misaligned attention activation, particularly in the presence of complex subsurface clutter. In contrast, the enhanced ACF-YOLO model, which integrates AFPN and CGAttention modules, produces sharply focused and spatially coherent attention maps that accurately correspond to the true target regions. This marked improvement in attention localization demonstrates the model’s superior capacity to discriminate weak target signals from heterogeneous background interference, thereby reinforcing the theoretical advantages of the proposed adaptive attention design. Such visualization evidence provides a compelling complement to quantitative results, affirming the robustness and practical applicability of the model in challenging ground-penetrating radar scenarios.

## 5. Results and Discussion

In this section, a comprehensive experimental evaluation and analysis of the performance of the improved model is conducted. The dataset is split into training and testing sets at a ratio of 8:2, with the data acquisition and construction process detailed in Section 3. The experimental section is structured into five parts: First, the specific training settings are described, including hyperparameter configuration, loss function design, and the evaluation metrics employed. Second, the recognition results of the improved model on the FKS-GPR testing set constructed in this study are analyzed in detail. Third, comparative experiments with several existing methods are performed to assess the performance advantages of the proposed approach from multiple perspectives. Fourth, to verify the model’s transferability and robustness, public datasets are introduced to evaluate its generalization capability. Finally, module-level ablation studies are conducted to examine the individual contributions and underlying mechanisms of each improved component in enhancing detection performance.

### 5.1. Network Training and Evaluation Metrics

The FKS-GPR dataset was divided into training and testing sets in an 8:2 ratio. All images were normalized and resized to a uniform grayscale format of 640 × 256 before being fed into the model. The entire training process was conducted under the PyTorch 1.13.1 framework, utilizing a 12-core Intel^®^ Xeon^®^ Silver 4310 CPU (Intel Corporation, CA, USA) with 128 GB of RAM and a base frequency of 2.1 GHz, along with an NVIDIA A100 GPU (NVIDIA Corporation, CA, USA). The network was optimized using the ADAM optimizer, and a cosine annealing learning rate scheduler was employed to dynamically adjust the learning rate, thereby improving convergence stability and training efficiency. The total number of training [21] epochs was set to 200, with a batch size of 16. To enhance the model’s discriminative capability under class imbalance scenarios, a weighted loss function was designed by linearly combining the standard Cross Entropy Loss with the Focal Loss. The formulation is given by Equation (7) as follows:

(7) $$L_{total}=\lambda \cdot L_{CE}+\left(1-\lambda \right)\cdot L_{Focal}$$

Here, $L_{CE}$ represents the standard Cross Entropy Loss, which focuses on ensuring the overall classification accuracy by penalizing incorrect predictions equally across all classes. In contrast, $L_{Focal}$ is the Focal Loss, which introduces a modulating factor to reduce the contribution of well-classified samples and instead emphasizes learning from hard-to-classify or minority class samples. This is particularly important in the context of ground-penetrating radar (GPR) images, where severely loosened regions or small-sized defects are often underrepresented and prone to misclassification. The balancing coefficient λ, empirically set to 0.5 in this study, is used to control the trade-off between the two loss components. By adjusting this parameter, the model can maintain a balance between general classification performance and its sensitivity to hard samples and rare classes.

Therefore, the total loss $L_{total}$ serves as an adaptive learning objective that not only drives the model to achieve higher overall detection accuracy but also effectively mitigates the negative impact of class imbalance by giving more attention to minority or low-confidence samples. This joint optimization strategy ultimately enhances the model’s robustness and reliability in real-world defect detection tasks.

To comprehensively and accurately evaluate the model’s performance in detecting voids and loosened targets in ground-penetrating radar images, five commonly used object detection metrics are employed: precision, recall, mean Average Precision (mAP), and GFLOPs [22,23]. Precision and recall are calculated as shown in Equations (8) and (9), respectively:

(8) $$\mathrm{Precision}=\frac{TP}{TP+FP}$$

(9) $$\mathrm{Recall}=\frac{TP}{TP+FN}$$

Mean Average Precision (mAP) is used to evaluate the overall performance of the multi-object detection model and is computed as described in Equations (10) and (11). The *F*_1_-score, which measures the balance between precision and recall, is defined in Equation (12). Additionally, GFLOPs represent the computational complexity of the neural network and are calculated according to Equation (13). Generally, higher mAP and *F*_1_-score indicate better model performance, while higher GFLOPs indicate greater computational capacity. The equations are as follows:

(10) $$\mathrm{AP}=\int_{0}^{1}\mathrm{Precision}\times \mathrm{RecalldRecall}$$

(11) $$\mathrm{mAP}=\frac{1}{\kappa }\underset{k\in K}{\Sigma }\mathrm{AP}(k)$$

(12) $$F_{1}=\frac{2\times \mathrm{Precision}\times \mathrm{Recall}}{\mathrm{Precision}+\mathrm{Recall}}$$

(13) $$\mathrm{GFLOPs}=\frac{\mathrm{Floating}-\mathrm{point}\;\mathrm{opertion}\;}{\mathrm{Run}\;\mathrm{time}\;\mathrm{in}\;\mathrm{sceonds}\;\times \;10^{9}}$$

where *TP* denotes the total number of true positive detections, *FP* is the total number of false positive detections, and *FN* is the total number of false negative detections. *K* represents the total number of classes contained in the dataset, and *k* refers to an individual category.

### 5.2. Validation of the Proposed Dataset—FKS-GPR

To validate the target detection performance and generalization capability of the proposed improved model in practical complex scenarios, a comprehensive evaluation was conducted based on the independently collected and precisely annotated FKS-GPR dataset. This dataset encompasses various road structure types, complex background interferences, and typical subsurface defects such as voids and severe looseness. It offers advantages including diverse sample distribution, high annotation accuracy, and strong representativeness, thereby realistically simulating the diverse challenges encountered in current ground-penetrating radar (GPR) applications.

During the construction of the test set, multiple B-scan radar images from real road sections were carefully selected. These images not only contain typical void and severe looseness targets but also retain complex background interference samples such as underground pipelines, interlayer cracks, and irregular reflections. This selection facilitates thorough validation of the model’s robustness and adaptability under multi-interference conditions.

**Figure 12:**
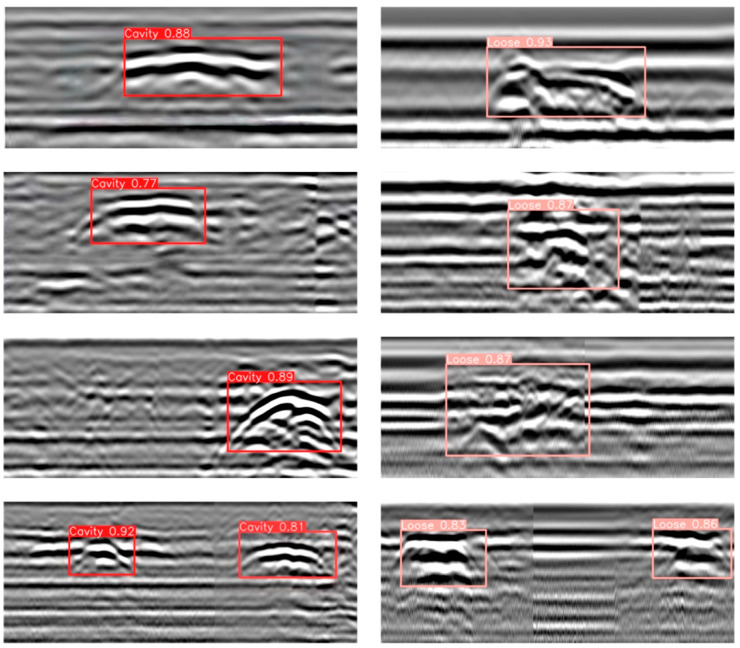
Visualization of the results.

In the detection experiments, the improved model trained as described in Section 5.1 was directly applied to perform inference and evaluation on the test set images. To assess the model’s performance from multiple perspectives, analyses were carried out focusing on three aspects: target localization accuracy, class discrimination ability, and consistency in responding to texture detail features. Qualitative visual results are shown in Figure 12, where the improved model demonstrates excellent detection performance across different types of anomalous regions. Specifically, for void targets, the model accurately extracts periodic reflection features between the upper and lower boundaries and achieves well-aligned detection bounding boxes. For severely loosened regions, the model effectively captures “cloud-like” texture disturbances and weak waveform reflection features, exhibiting strong classification confidence and discriminative capability. In particular, as shown in Figure 12, the model achieves consistently high detection confidence scores for both voids and severe looseness targets. For cavity defects, the highest confidence score reaches 0.92, with the lowest at 0.77, and the average remains above 0.80. For looseness defects, the highest confidence reaches 0.93, with the lowest at 0.83. These results further confirm the model’s effective capability to distinguish between different types of subsurface defects under complex background interference. Notably, even in complex scenarios with irregular target shapes, closely adjacent multiple targets, and significant background clutter, the model maintains high detection stability and robustness, demonstrating strong practical engineering adaptability. Furthermore, a quantitative evaluation of the detection results on the test set was performed using metrics including precision, recall, and mAP. As shown in Table 2, the proposed improved model outperforms the original YOLOv8 baseline across all key metrics, fully validating the effectiveness of the structural improvements in enhancing the recognition of voids and severe looseness targets in ground-penetrating radar images.

### 5.3. Model Comparison Experiments

To further validate the performance advantages of the proposed improved model in the task of road defect detection, four representative mainstream object detection algorithms were selected as baseline comparisons: Faster R-CNN (a typical two-stage detection method) [24], YOLOv8 (a widely adopted lightweight detection framework), YOLOv11 (a newly released single-stage detection model) [25], and the recently introduced EfficientDet-D2 detection method [26]. These models, along with the proposed improved ACF-YOLO model, were systematically evaluated on the constructed FKS-GPR test set under a unified training strategy and parameter configuration, ensuring the fairness and comparability of the experimental results.

**Figure 13:**
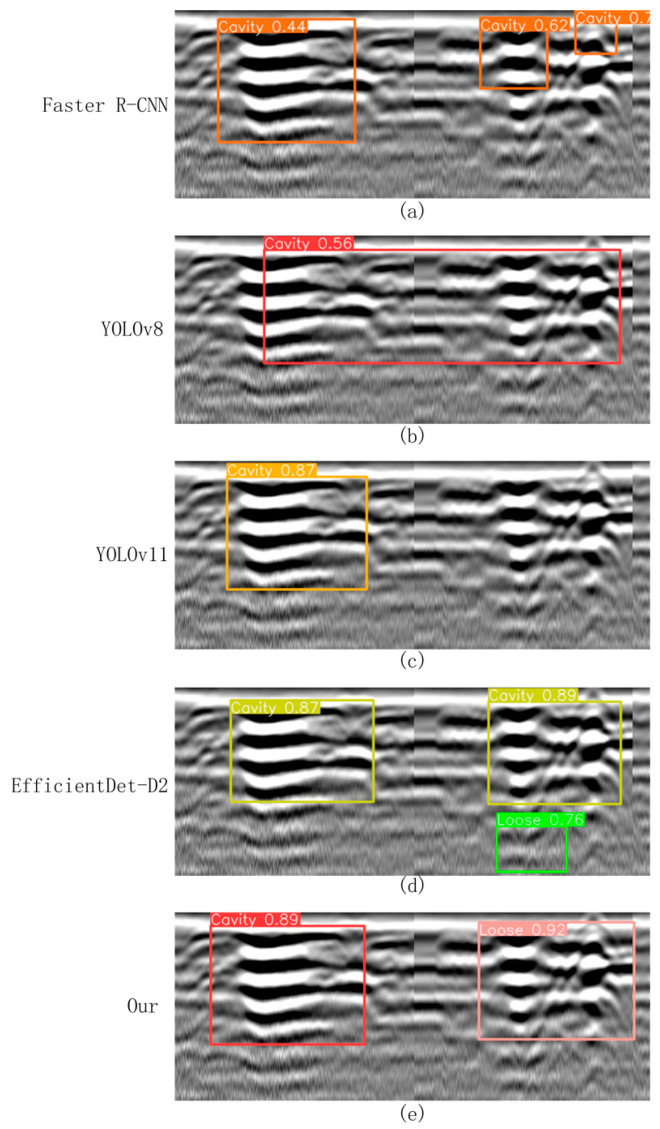
Visual comparison of detection results across models. (**a**) Detection results of Faster R-CNN; (**b**) detection results of YOLOv8; (**c**) detection results of YOLOv11; (**d**) detection results of EfficientDet-D2; and (**e**) detection results of the proposed ACF-YOLO model.

This comparative experiment focuses on two typical types of subsurface defects: “cavities” and “severely loosened” regions. The performance of the five models was comprehensively assessed in terms of detection of precision, recall, *F*_1_-score, and model complexity (measured in GFLOPs). The detailed quantitative results are summarized in Table 2, while representative visual detection results are presented in Figure 13. Specifically, Figure 13a shows the detection results of Faster R-CNN under the same testing conditions, while Figure 13b–e display the detection results of YOLOv8, YOLOv11, EfficientDet-D2, and the proposed ACF-YOLO model, respectively.

Faster R-CNN (Figure 13a), a classical two-stage detection model, demonstrated strong feature extraction capabilities. However, due to its complex network architecture and high sensitivity to background interference, it exhibited a relatively high false detection rate. Specifically, in B-scan images, non-target structures such as cracks and underground pipelines were often misclassified as cavities or severely loosened areas. As a result, the detection accuracies for cavities and loosened regions were only 86.2% and 81.6%, respectively, with an overall average *F*_1_-score of approximately 81.9%. In addition, the high computational cost of this model (18.5 GFLOPs) further limits its potential for real-time applications. In particular, Faster R-CNN exhibited inference latencies of 74 ms on GPU and 329 ms on CPU, corresponding to 13.5 FPS and 3.0 FPS, with memory usage reaching 7.8 GB (GPU) and 4.9 GB (CPU), which is significantly higher than other models and further constrains its real-time applicability and resource efficiency.

As a lightweight single-stage detection model, YOLOv8 (Figure 13b) achieved detection precisions of 88.6% and 83.2% for cavities and loosened areas, with corresponding recall rates of 86.7% and 82.1%, and an average *F*_1_-score of 85.1%, all while maintaining a relatively low computational burden (7.3 GFLOPs). However, its performance in modeling target boundary details remains limited. In particular, when handling adjacent or visually similar targets, YOLOv8 tends to produce overlapping detection boxes and class confusion, which can compromise detection stability and accuracy. In terms of deployment performance, YOLOv8 achieved inference latencies of 15 ms (GPU) and 97 ms (CPU), corresponding to 66.7 FPS and 10.3 FPS, with moderate memory usage of 4.1 GB (GPU) and 2.5 GB (CPU), offering a favorable balance between detection performance and computational efficiency.

In contrast, YOLOv11 (Figure 13c) integrates a deeper feature extraction network and an optimized anchor box mechanism, significantly enhancing its boundary recognition and class discrimination capabilities. It achieved detection precisions exceeding 90% for both cavity and loosened targets, with an average *F*_1_-score of 89.3%. YOLOv11 demonstrated superior generalization ability in complex backgrounds with strong interference, outperforming the aforementioned models. Nonetheless, it still suffers from occasional missed and false detections, particularly when dealing with highly complex background textures. Deployment evaluation shows that YOLOv11 achieved inference latencies of 18 ms (GPU) and 108 ms (CPU), with corresponding FPS of 55.5 and 9.3, and memory usage of 4.6 GB (GPU) and 2.6 GB (CPU), achieving a good balance between detection accuracy and resource consumption.

This study also reproduced and systematically evaluated the EfficientDet-D2 model (Figure 13d). The results show that it achieved a detection precision of 90.0% and a recall of 87.5% for cavity detection, while for loosened soil, the precision and recall reached 87.4% and 83.1%, respectively. Benefiting from its compound multi-scale feature fusion mechanism and lightweight architecture (only 1.5 GFLOPs), EfficientDet-D2 demonstrated high accuracy and stability when identifying cavities with clear boundaries and regular shapes. However, when dealing with ambiguous boundaries, low-contrast features, and complex background clutter, the model tended to misclassify severely loosened regions as cavities, resulting in decreased classification accuracy. Its inference latency was 22 ms on GPU and 121 ms on CPU, with corresponding FPS of 45.5 and 8.2, and memory usage of 3.0 GB (GPU) and 2.0 GB (CPU), highlighting its computational efficiency despite slightly reduced detection robustness compared to the top-performing models.

In contrast, the proposed improved ACF-YOLO model (Figure 13e) maintains a lightweight computational load (9.2 GFLOPs) while integrating an adaptive multi-scale feature focusing mechanism and multi-level attention modules, significantly enhancing its sensitivity to weak boundaries and low-echo targets. The experimental results demonstrate that the model achieves detection precisions of 93.1% and 91.8% for cavities and loosened soil, respectively, with corresponding recall rates of 91.0% and 90.6%, and an average *F*_1_-score as high as 91.6%, substantially outperforming all other benchmark models. The proposed model not only improves detection robustness under complex textured backgrounds but also maintains high computational efficiency, indicating strong applicability and deployment potential in practical engineering scenarios. Deployment evaluation further confirms its practicality: the ACF-YOLO model achieved inference latencies of 19 ms (GPU) and 113 ms (CPU), corresponding to FPS of 52.6 and 8.8, with memory usage of 5.2 GB (GPU) and 2.7 GB (CPU), effectively balancing detection accuracy with resource utilization and real-time performance.

### 5.4. Validation on Public Datasets

**Figure 14:**
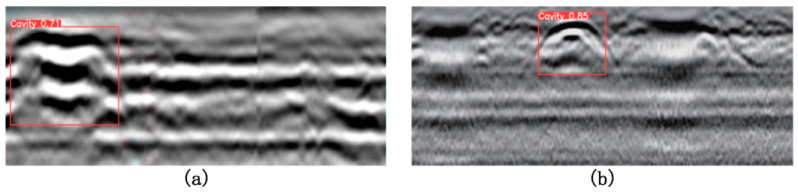
Visualization of detection results on public datasets. (**a**) B-scan from a real roadbed void detection dataset provided by the research team at Shenyang Jianzhu University, representing a typical urban road environment. (**b**) B-scan from a void detection dataset collected on-site in Quzhou, Zhejiang Province, using the LTD-2600 GPR system, as described in Reference [27].

To further verify the generalization capability of the proposed model and its adaptability in practical applications, this section evaluates the model on two representative public ground-penetrating radar (GPR) void detection datasets, shown in Figure 14a,b, corresponding to References [27,28], respectively. The dataset in Figure 14a was collected from an actual roadbed void detection task at Shenyang Jianzhu University, representing a typical urban road environment. This dataset features complex background interference, diverse underground structures, and realistic target echo characteristics, effectively reflecting the model’s robustness and detection capability under various interference signals and echo intensity variations. The dataset in Figure 14b originates from a vehicle lane detection task on Beimen Street in Kecheng District, Quzhou City, Zhejiang Province (covering the section from Xi’an Road to Xinheyan Road), collected on-site using the LTD-2600 GPR system (Changchun Technology Co., Changchun, China) equipped with a CG 270 MHz antenna (Geophysical Survey Systems Inc. (GSSI), NH, USA). The radar profiles in this dataset contain prominent multiple reflections and strong signal interference, with blurred target boundaries and complex texture structures. Therefore, it serves as a more challenging benchmark for assessing the model’s detection accuracy and fault tolerance in high-noise environments.

The detection results on the dataset in Figure 14a demonstrate that the proposed model can accurately locate and identify key reflective features of void regions. Even under conditions of multi-target interference and weak echo signals, the model effectively suppresses background clutter, and the predicted bounding boxes closely align with the true target boundaries. This indicates strong discriminative ability when handling complex textures and signal overlaps, with low overall false positive and false negative rates, showcasing excellent practical applicability. The results in Figure 14b further validate the model’s robustness against multiple reflections and complex interference backgrounds. Although the voids in this scenario show subtle differences from surrounding structures, posing a typical “weak target” detection challenge, the model still successfully captures key reflection patterns and provides reasonably accurate target localization. While some detection errors occur in areas with blurred boundaries, the overall performance sufficiently demonstrates the model’s adaptability and practical potential in high-noise, high-complexity environments.

It is worth noting that currently available public ground-penetrating radar datasets primarily focus on strong reflection targets such as cavities and pipelines, lacking dedicated data support for areas of severe soil loosening. Such targets exhibit weak reflected signals, blurred boundaries, and irregular textures in radar images, making their detection challenging. Existing datasets are insufficient to capture the diversity and complexity of these targets. To address this limitation in the validation framework, this study conducted field surveys and data collection specifically targeting severely loosened regions, thereby constructing a dedicated independent test set. This dataset provides a more targeted data foundation and experimental support for subsequent model performance evaluation and algorithm optimization in weak target detection tasks.

### 5.5. Ablation Study

To systematically evaluate the individual contributions of each proposed module to the overall model performance, a series of ablation experiments were conducted on the FKS-GPR test set. The AFPN, AKConv, and CGAttention modules were progressively removed to construct a set of comparative models based on the YOLOv8 framework. For each model, key performance metrics—including mean Average Precision (mAP), mean recall, number of parameters (Params) [29], computational complexity (GFLOPs), inference latency, runtime FPS on GPU/CPU, and memory usage—were comprehensively analyzed for both “cavity” and “severely loosened soil” target detection tasks.

The results demonstrate that the introduction of the AFPN module alone (Model B) slightly increased the computational complexity from 7.3 GFLOPs to 7.8 GFLOPs (a 6.8% rise). Meanwhile, the detection accuracy (mAP) and recall improved by 1.5 and 1.4 percentage points, respectively. This performance gain is primarily attributed to the module’s multi-scale feature fusion and cross-layer attention mechanisms, which significantly enhance the detection of small and weak targets, especially in the presence of complex background interference. In terms of deployment, Model B maintained real-time feasibility with inference latencies of 16 ms (GPU) and 101 ms (CPU), corresponding to 62.5 FPS and 9.9 FPS, and moderate memory consumption (4.3 GB GPU, 2.6 GB CPU).

The AKConv module (Model C) further enhanced boundary modeling capabilities, increasing GFLOPs to 8.0 (a 9.6% increase) and achieving additional mAP and recall improvements of 2.0 and 1.6 percentage points, respectively. Its dynamic convolution kernel structure adaptively captured top-edge reflection features, effectively addressing the challenge of significant boundary variations between cavities and loosened regions. Deployment evaluation revealed inference latencies of 17 ms (GPU) and 104 ms (CPU), FPS of 58.8 and 9.6, and memory usage of 4.5 GB (GPU) and 2.6 GB (CPU), demonstrating a favorable trade-off between detection accuracy and computational efficiency.

To further verify the effectiveness and superiority of the AKConv module, a comparative ablation experiment was conducted by replacing AKConv with the widely used Deformable Convolutional Networks v2 (DCNv2) to create Model C2. As shown in Table 3, although DCNv2 (Model C2) provided some performance improvements over the baseline YOLOv8 (mAP of 86.5%, recall of 85.5%), it still lagged behind the AKConv-based Model C in both detection accuracy and robustness. Critically, Model C2 incurred a substantial increase in computational complexity, with parameters rising to 13.6 M and GFLOPs to 10.5. Moreover, Model C2 exhibited higher inference latency (21 ms GPU, 118 ms CPU) and lower processing speeds (47.6 FPS GPU, 8.4 FPS CPU), along with increased memory consumption (5.5 GB GPU, 2.8 GB CPU). These findings further underscore the computational efficiency advantage of the proposed AKConv module.

The underlying reason for this performance gap lies in the distinct design motivations of the two modules. DCNv2 was originally developed for natural image analysis, aiming to improve adaptability to geometric transformations and object contour variations in tasks characterized by visually prominent boundaries. However, it was not specifically optimized for the unique reflection patterns and boundary characteristics of ground-penetrating radar (GPR) imagery, where targets such as cavities typically generate strong reflections with sharp boundaries, while severely loosened soil regions produce weak echoes with blurred edges and gradual grayscale transitions. Without dedicated modeling of these signal intensity variations, DCNv2 is more susceptible to missed detections and false positives, particularly when dealing with weak boundary targets.

In contrast, the proposed AKConv module is specifically tailored to the physical properties of GPR images. By dynamically adjusting sampling positions and receptive field sizes based on local structural features, AKConv enhances sensitivity to both strong boundary reflections and weakly defined targets. Furthermore, by constraining deformation flexibility within a controlled range, AKConv significantly reduces computational complexity while maintaining robust feature extraction. This design ensures real-time performance and stability, making it highly suitable for practical engineering deployments.

These quantitative findings further validate the theoretical basis outlined in Section 4.3. The AKConv module not only outperforms DCNv2 in detection accuracy but also exhibits superior computational efficiency and robustness in complex, noisy underground environments. Its hybrid adaptive mechanism simultaneously strengthens the network’s perception of both high-gradient (clear boundary) and low-gradient (blurred boundary) regions, substantially improving detection accuracy and generalization across multiple types of GPR subsurface anomalies.

The CGAttention module (Model D) focused on enhancing the perception of echo textures and local morphological patterns. Its inclusion led to an increase in GFLOPs to 7.7, with corresponding improvements in mAP and recall by 1.1 and 0.8 percentage points, respectively. By integrating both channel and spatial attention mechanisms, CGAttention effectively captured anomalous texture regions and improved the discrimination of weak, disordered reflections typically associated with severely loosened soils. Deployment evaluation showed stable computational performance with inference latencies of 16 ms (GPU) and 99 ms (CPU), FPS of 62.5 and 10.1, and memory consumption of 4.2 GB (GPU) and 2.5 GB (CPU).

To explore the complementary benefits of multi-module integration, three intermediate models (Models E, F, and G) combining different subsets of the proposed modules were also evaluated. These models consistently achieved performance improvements over the baseline while maintaining manageable computational overhead. For instance, Model E achieved inference latencies of 18 ms (GPU) and 108 ms (CPU), with processing speeds of 55.5 FPS and 9.3 FPS, and moderate memory consumption (4.8 GB GPU, 2.7 GB CPU). These results demonstrate that multi-module integration can effectively enhance detection performance without causing excessive computational burden.

Ultimately, the fully integrated model (Model H), combining AFPN, AKConv, and CGAttention, delivered the best overall performance. The model achieved a mAP of 92.2% and a recall of 90.8%, representing improvements of 6.6 and 6.4 percentage points over the baseline YOLOv8. Although the computational cost increased to 9.2 GFLOPs (a 26% rise) and parameters grew to 13.8 M, the model maintained a lightweight structure suitable for edge deployment and real-time applications. Deployment evaluation confirmed the practicality of Model H, with inference latencies of 19 ms (GPU) and 113 ms (CPU), FPS of 52.6 and 8.8, and memory consumption of 5.2 GB (GPU) and 2.7 GB (CPU), highlighting its balanced performance across accuracy, speed, and resource efficiency.

In summary, the AFPN, AKConv, and CGAttention modules address the complex reflection characteristics of cavity and severely loosened soil targets from three complementary perspectives: feature fusion, boundary modeling, and texture perception. Their multidimensional collaborative modeling strategy significantly enhances detection accuracy and robustness while maintaining a reasonable and controllable computational load, serving as a key driver of overall model performance improvement.

### 5.6. Statistical Significance Analysis of Detection Performance

To further verify the reliability and robustness of the reported performance improvements, we conducted a statistical significance analysis based on repeated experiments. Specifically, each detection model was independently trained and evaluated five times on the FKS-GPR test set under identical training conditions. For each model, we computed the mean (μ) and standard deviation (σ) of key evaluation metrics—mean Average Precision (mAP), average recall, and average *F*_1_-score—to assess stability across runs.

To determine whether the performance gains achieved by the proposed ACF-YOLO model were statistically significant rather than due to random fluctuations, we performed two-tailed paired *t*-tests comparing the mAP results of ACF-YOLO against those of the other baseline models. A significance threshold of *p* < 0.05 was adopted.

Table 4 presents the detailed results. The proposed ACF-YOLO model consistently achieved the highest average scores across all metrics, while maintaining low standard deviations, reflecting its strong robustness and stability. More importantly, all *p*-values were below the 0.05 threshold, confirming that the improvements over YOLOv8, Faster R-CNN, YOLOv11, and EfficientDet-D2 are statistically significant. These findings reinforce the validity of the proposed method and address potential concerns about performance variability arising from random initialization, data partitioning, or stochastic optimization. The consistent superiority and statistical significance of ACF-YOLO demonstrate its generalization capability and reliability in real-world GPR detection scenarios.

The results of this section further validate the effectiveness and reliability of the proposed ACF-YOLO model. Its consistently superior detection accuracy, combined with low variability and statistically significant improvements over other models, confirms that the observed gains stem from the carefully designed adaptive feature fusion and attention mechanisms—rather than random chance—thus enhancing the credibility of the method for engineering deployment in complex ground-penetrating radar scenarios.

## 6. Conclusions

This study addresses the challenge of automatically identifying road voids and severe looseness in ground-penetrating radar (GPR) B-scan images by proposing a recognition method based on multidimensional image feature differences. By systematically analyzing three key dimensions—texture structure, intensity and clarity of the top reflective interface, and the regularity of internal reflection waveforms—a robust discriminative framework was developed to accurately differentiate between voids and severely loosened areas. Based on this framework, a high-fidelity and well-annotated real-world dataset, FKS-GPR, was constructed, significantly enhancing data authenticity and representativeness and filling the current research gap dominated by simulated data. An improved ACF-YOLO network, incorporating multiple advanced modules, was designed and implemented, demonstrating notable improvements in detection accuracy and robustness on this dataset. Extensive experimental validation confirms the superior performance of the proposed method in recognizing complex underground defects, underscoring the effectiveness and practical value of the multidimensional image feature discrimination approach. Future work will focus on expanding field data collection across diverse scenarios and constructing a dedicated test set for weak targets, such as severe looseness, to further facilitate fine-grained evaluation and targeted model optimization.

This research not only offers a viable technical solution for the automatic identification of complex underground defects in GPR imagery but also lays a solid foundation in data and methodology for road maintenance and safety assessment.

We would like to acknowledge that the FKS-GPR dataset, which was used in this study, is currently not publicly available due to licensing restrictions. We appreciate the support of the organizations involved in its creation and access, and we hope to provide a future update regarding its availability. We apologize for any inconvenience this may cause and encourage researchers to stay tuned for future updates on data access.

## Figures and Tables

**Table 1:** Attributes of the dataset.

Types of Data Augmentation	Implementation Details	Number of B-Scans	FKS-GPR
Rotation and Flipping	Random rotation and horizontal/vertical flipping on all 350 images.	350	2800
Brightness Disturbance	Brightness adjustment was randomly applied to half of the 350 images (including both original and flipped images), with three different intensity levels set: +15%, −15%, and +30%.	525	
Local Contrast	A different set of 175 images (the half not subjected to brightness adjustment) was selected for contrast enhancement, with three different intensity levels applied.	525	
Image Cropping	Two additional crops were performed on each of the 350 original B-scan images from angles different from the original. The cropping positions were randomly selected from the top-left, top-right, bottom-left, or bottom-right corners, ensuring that they differ from the original B-scan.	700	
Noise Addition	The 350 original images were split into two halves, with Gaussian noise added to one half and salt-and-pepper noise added to the other half.	350	
Original B-scan	No	350	

**Table 2:** Comparison of performance with other algorithms.

Model Name	Cavity Precision	Cavity Recall	Loose Precision	Loose Recall	mAP/%	Average *F*_1_	GFLOPs	Inference Latency (ms) (GPU/CPU)	FPS (GPU/CPU)	Memory Usage (GB) (GPU/CPU)
YOLOv8	88.6	86.7	83.2	82.1	85.6	85.1	7.3	15/97	66.7/10.3	4.1/2.5
Faster R-CNN	86.2	82.4	81.6	77.9	82.1	81.9	18.5	74/329	13.5/3.0	7.8/4.9
YOLOv11	90.8	89.5	90.1	86.9	90.4	89.3	8.9	18/108	55.5/9.3	4.6/2.6
EfficientDet-D2	90.0	87.5	87.4	83.1	89.2	87.0	1.5	22/121	45.5/8.2	3.0/2.0
Our work	93.1	91.0	91.8	90.6	92.2	91.6	9.2	19/113	52.6/8.8	5.2/2.7

**Table 3:** Comparison of object detection performance in the ablation study.

Model ID	YOLOv8	AFPN	AKConv	DCNv2	CGAttention	mAP/%	Average Recall	Params (M)	GFLOPs	Inference Latency (ms) (GPU/CPU)	FPS (GPU/CPU)	Memory Usage (GB) (GPU/CPU)
A	√					85.6	84.4	11.2	7.3	15/97	66.7/10.3	4.1/2.5
B	√	√				87.1	85.8	12.0	7.8	16/101	62.5/9.9	4.3/2.6
C	√		√			87.6	86.0	11.8	8.0	17/104	58.8/9.6	4.5/2.6
C2	√			√		86.5	85.5	13.6	10.5	21/118	47.6/8.4	5.5/2.8
D	√				√	86.7	85.2	11.6	7.7	16/99	62.5/10.1	4.2/2.5
E	√	√	√			89.4	87.9	12.7	8.3	18/108	55.5/9.3	4.8/2.7
F	√	√			√	88.8	87.1	12.4	8.2	18/107	55.5/9.3	4.7/2.7
G	√		√		√	89.7	88.2	12.2	8.4	18/108	55.5/9.3	4.8/2.7
H	√	√	√		√	92.2	90.8	13.8	9.2	19/113	52.6/8.8	5.2/2.7

**Table 4:** Statistical analysis of detection performance over five independent runs.

Model Name	mAP (%) (μ ± σ)	Average Recall (%) (μ ± σ)	Average *F*_1_ (%) (μ ± σ)	*p*-Value (vs. ACF-YOLO)
YOLOv8	85.5 ± 0.21	84.3 ± 0.25	85.0 ± 0.23	*p* = 0.002
Faster R-CNN	82.0 ± 0.30	80.6 ± 0.34	81.7 ± 0.28	*p* = 0.001
YOLOv11	90.3 ± 0.18	88.7 ± 0.20	89.2 ± 0.19	*p* = 0.015
EfficientDet-D2	89.1 ± 0.25	86.5 ± 0.27	86.9 ± 0.24	*p* = 0.008
Our work	92.1 ± 0.14	90.7 ± 0.17	91.5 ± 0.15	—

Note: The *p*-values were obtained via two-tailed paired *t*-tests on mAP values comparing each model against ACF-YOLO. Significant differences are denoted by *p* < 0.05.

## Data Availability

The data supporting the findings of this study are not publicly available due to privacy and ethical restrictions.
